# Dynamic transcriptomic m^6^A decoration: writers, erasers, readers and functions in RNA metabolism

**DOI:** 10.1038/s41422-018-0040-8

**Published:** 2018-05-22

**Authors:** Ying Yang, Phillip J. Hsu, Yu-Sheng Chen, Yun-Gui Yang

**Affiliations:** 1 CAS Key Laboratory of Genomic and Precision Medicine, Collaborative Innovation Center of Genetics and Development, College of Future Technology, Beijing Institute of Genomics, Chinese Academy of Sciences, Beijing, 100101 China; 2 University of Chinese Academy of Sciences, Beijing, 100049 China; 3 Institute of Stem Cell and Regeneration, Chinese Academy of Sciences, Beijing, 100101 China; 4 Department of Chemistry and Institute for Biophysical Dynamics, Howard Hughes Medical Institute, The University of Chicago, Chicago, IL 60637 USA; 5 Medical Scientist Training Program/Committee on Immunology, The University of Chicago, Chicago, IL 60637 USA

## Abstract

*N*^6^-methyladenosine (m^6^A) is a chemical modification present in multiple RNA species, being most abundant in mRNAs. Studies on enzymes or factors that catalyze, recognize, and remove m^6^A have revealed its comprehensive roles in almost every aspect of mRNA metabolism, as well as in a variety of physiological processes. This review describes the current understanding of the m^6^A modification, particularly the functions of its writers, erasers, readers in RNA metabolism, with an emphasis on its role in regulating the isoform dosage of mRNAs.

## Introduction

Over 150 RNA modifications have been identified as post-transcriptional regulatory marks in multiple RNA species, including messenger RNAs (mRNAs), transfer RNAs (tRNAs), ribosomal RNAs (rRNAs), small non-coding RNAs, and long non-coding RNAs (lncRNAs).^1–4^ These modifications regulate several facets of RNA processing or metabolism, including alternative splicing,^5–15^ export,^16–18^ stability,^19–25^ and translation.^26–32^ *N*^6^-methyladenosine (m^6^A) is the most prevalent modification in the mRNA of many eukaryotic species, including yeast,^33–35^ plants,^36–38^ flies,^39^ and mammals.^40–47^ Although it was first discovered in the 1970s,^44,48–50^ detailed studies of its functions did not begin until around 2012, when transcriptome-wide profiling of m^6^A was made possible through antibody-based immunoprecipitation followed by high-throughput sequencing. More than 10,000 m^6^A peaks have been validated in over 25% of human transcripts. It was found to be on a consensus RNA motif of RRACH (R = A or G; H = A, U, or C), and enriched in long exons, near stop codons and 3’ untranslated regions (3’ UTRs).^51,52^

**Fig. 1:**
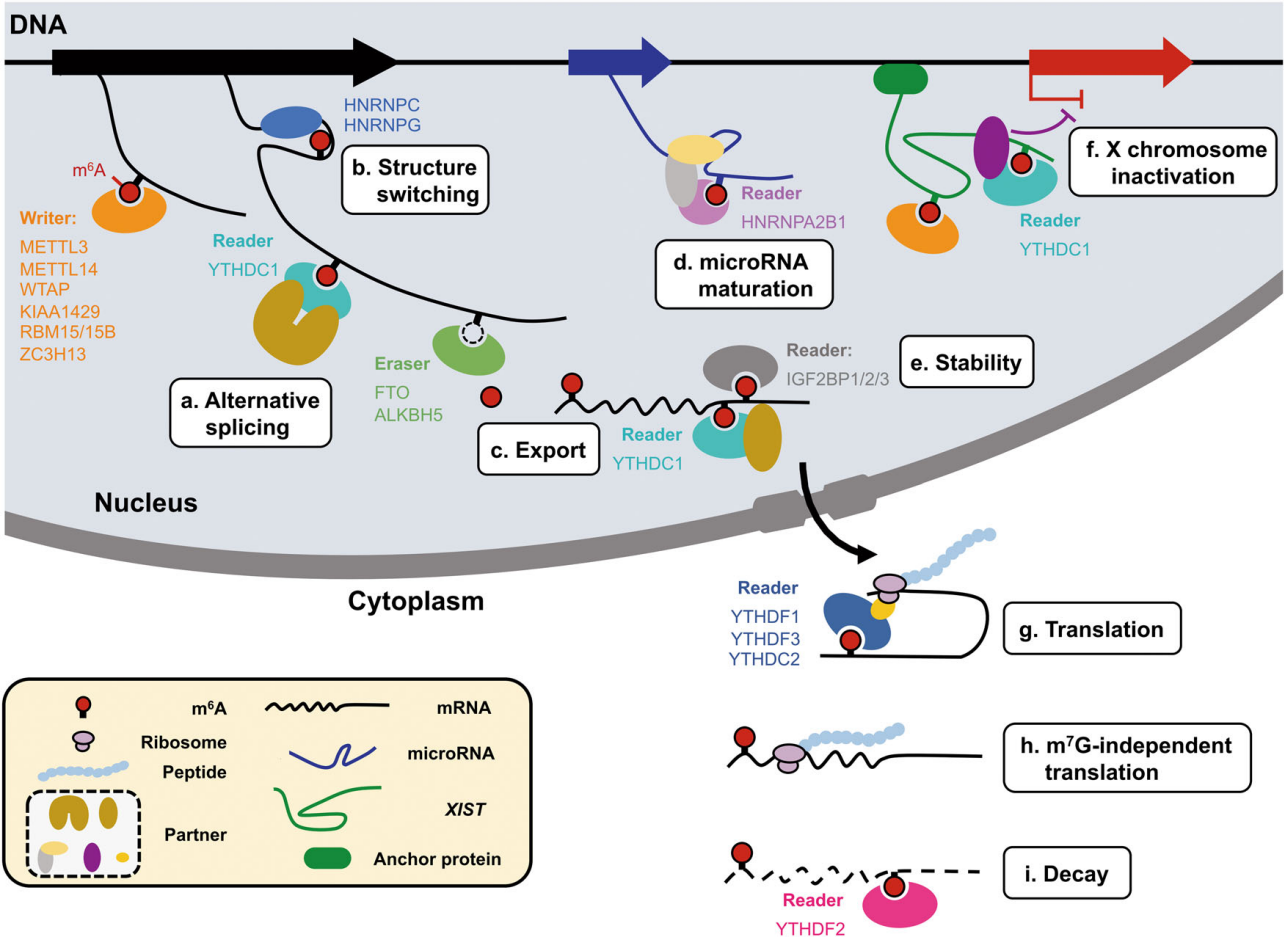
Diverse molecular functions of m^6^A. In eukaryotic cells, RNA m^6^A level is dynamically regulated by “writers” and “erasers”, and recognized by “readers” in direct or indirect ways. The diversity of cellular processes involving m^6^A is mainly contributed by various “readers”. The nuclear m^6^A modulates a mRNA alternative splicing,^5,7–9,11–15,54,58–60^ b secondary structure switching,^5,11,105^ **c** mRNA export,^17,18^ d pri-miRNA processing,^104,135^ e mRNA stability^24^ and f *XIST*-dependent X chromosome inactivation,^57^ while the cytoplasmic m^6^A (g and h) enhances mRNA translation efficiency^26,27,29,32,106^ and i accelerates mRNA decay.^20,21,23,108^

The abundance and effects of m^6^A on RNA are determined by the dynamic interplay between its methyltransferases (“writers”), binding proteins (“readers”), and demethylases (“erasers”) (Fig. 1). In this review, we provide a comprehensive summary about the biological functions of m^6^A writers, readers, and erasers, as well as the role of m^6^A in splicing regulation.

## Methyltransferases/Writers

m^6^A is installed by a multicomponent methyltransferase complex consisting of Methyltransferase Like 3 (METTL3),^53^ METTL14,^13,22,54,55^ Wilms Tumor 1 Associated Protein (WTAP),^56^ KIAA1429,^22^ RNA Binding Motif Protein 15 (RBM15),^57^ and zinc finger CCCH domain-containing protein 13 (ZC3H13).^58–60^ METTL3 is the catalytic subunit, while METTL14 acts as the RNA-binding platform.^61–63^

### METTL3

Purified METTL3 protein selectively methylates the GAC and AAC sequences in synthetic single-stranded RNA *in vitro*.^15,64^ METTL3 was also observed to localize onto nuclear speckles enriched with mRNA splicing factors as shown by in vivo immunofluorescence analysis, indicating a potential regulatory role of m^6^A in mRNA metabolism.^13^ Early studies used nuclear lysate to perform the methyltransferase assay, supporting the nuclear activity of METTL3. However, these studies also detected the activity of METTL3 in the cytoplasmic fraction, although at a lower level than that in the nuclear lysate.^65^ One recent study showed that cytoplasmic METTL3 acts to promote translation independent of its methyltransferase activity.^66^

As the core methyltransferase subunit, METTL3 has been demonstrated to modulate embryonic development,^67,68^ cell reprogramming^47,68^ and spermatogenesis,^46^ while its deletion in mice causes early embryonic lethality.^69^ METTL3 also regulates T cell homeostasis^70^ and endothelial-to-haematopoietic transition^25,71^ via methylation of specific target transcripts.

METTL3 is a highly conserved protein, with homologs in multiple species including *Saccharomyces cerevisiae* (IME4), *Drosophila melanogaster* (IME4) and *Arabidopsis Thaliana* (MTA). ^33,36,39,67,72^ In *Arabidopsis thaliana*, deficiency of the METTL3 homolog MTA affects development and growth,^36,67^ while in *Saccharomyces cerevisiae*, IME4 plays an essential regulatory role during meiosis and sporulation.^33,35,73^ In *Drosophila*, mutation of IME4 impairs neuronal functions and influences sex determination by modulating female-specific splicing of Sex-lethal (*Sxl*) gene.^8,9^

### METTL14

METTL14 has been identified as another component of the m^6^A methyltransferase complex; both METTL3 and METTL14 are highly conserved in mammals, and form a stable heterodimer.^13,54,55^ Individually, METTL3 and METTL14 exhibit comparable weak methyltransferase activity in vitro. However, the METTL3-METTL14 complex has a much higher catalytic activity.^54,55^ A recent study on the crystal structure of the METTL3-METTL14 methyltransferase domain (MTD) complex illustrated that the primary function of METTL14 is not to catalyze methyl-group transfer but to offer an RNA-binding scaffold, allosterically activating and enhancing METTL3 the catalytic activity of METTL3.^61–63^ Two CCCH-type zinc finger domains (ZFDs) preceding the MTD in the N-terminus of METTL3 serve as the RNA target recognition domain.^74^

Similar to METTL3, METTL14 plays essential roles in diverse biological processes. Depletion of METTL14 causes a block in embryonic stem cell self-renewal and differentiation, embryonic developmental defects, and impaired gametogenesis in various organisms.^55,69,75^ METTL14 and METTL3 participate in neurogenesis by modulating cell-cycle progression of cortical neural progenitor cells in an m^6^A-dependent manner.^40^ METTL14 also plays a tumor-suppressor role in glioblastoma, and the depletion of METTL3 or METTL14 enhances growth and self-renewal of glioblastoma stem cells and promotes tumor progression.^76^ On the other hand, Weng et al. reported very recently that METTL14 is highly expressed in normal hematopoietic stem/progenitor cells and in various subtypes of acute myeloid leukemia (AML), and plays a critical oncogenic role in the development and maintenance of AML by blocking myeloid differentiation and promoting self-renewal of leukemia stem/initiating cells (LSCs/LICs).^77^ Thus, METTL14 might function in cancers in a tissue/cancer-type specific manner.

### WTAP

WTAP interacts with METTL3 and METTL14 to modulate the m^6^A levels of RNA transcripts.^13,36,54,56^ WTAP is a ubiquitously expressed nuclear protein that appears to play a role in both transcriptional and posttranscriptional regulation of certain cellular genes.^78^ As it lacks methyltransferase domains, WTAP shows no catalytic activity of m^6^A modification or effects on the activity of the METTL3-METTL14 complex in vitro, but it is required for their localization in nuclear speckles that are enriched with various precursor messenger RNA (pre-mRNA) processing factors.^13,54^

Depletion of WTAP induces tissue-dependent defects. Similar to zebrafish embryos depleted of METTL3, zebrafish embryos lacking WTAP undergo increased apoptosis.^13^ WTAP has been reported to be upregulated in > 30% AML cases and paly an oncogenic role in AML.^41^ Although it has not yet been investigated whether m^6^A is involved in the role of WTAP in AML, METTL14 expression was found to be increased in AML,^77^ implying that regulation of m^6^A levels by the m^6^A writer complex may be a key factor in AML oncogenesis.

### KIAA1429

KIAA1429 was identified as another component of the m^6^A methyltransferase complex by both proteomic screening and cellular studies.^22^ Its *Drosophila* ortholog interacts with *Drosophila* WTAP and regulates alternative splicing of pre-mRNAs involved in sex determination.^79,80^ Depletion of KIAA1429 induced an about 4-fold decrease in m^6^A peak scores in human A549 cells,^22^ suggesting an important regulatory role of KIAA1429 in the methyltransferase complex. In support of this, Yue et al. recently reported that VIRMA/KIAA1429 recruits the catalytic core components (METTL3/METTL14/WTAP) to guide region-selective m^6^A methylation.^81^

### RBM15/RBM15B

One recent study suggested that RBM15 and its paralogue RBM15B direct the methylation of adenosine residues in both mRNAs and the lncRNA target *XIST*. Immunoprecipitation analysis indicated that RBM15/RBM15B bind and recruit the WTAP-METTL3 complex to specific sites. Both RBM15 and RBM15B comprise three RNA recognition motif (RRM) domains, and combined analyses of iCLIP-seq data of RBM15/RBM15B and m^6^A-miCLIP-seq data revealed that RBM15/RBM15B binding sites are significantly enriched at the locations adjacent to m^6^A methylation sites.^57^ This study suggests a mechanism of selective activity of the methyltransferase towards *XIST*.

Consistently, the RBM15 homolog, Spenito (Nito), was shown to be a novel subunit of the methyltransferase complex required for m^6^A formation in mRNAs in *Drosophila*.^8^

### ZC3H13

Most recently, there are three studies demonstrating that ZC3H13 is another component of m^6^A writer complex and regulates m^6^A methylation.^58–60^ Wen *et al*. revealed that mouse Zc3h13 is required for nuclear localization of the Zc3h13-WTAP-Virilizer-Hakai complex and regulates mouse embryonic stem cell (ESC) self-renewal through facilitating m^6^A methylation.^58^ Knuckles et al. showed that mouse Zc3h13 and its fly homolog, CG7358 (named as *Flacc*), serve as an adaptor between RBM15/Nito and WTAP/Fl(2)d, bridging RBM15/Nito to the m^6^A machinery to promote m^6^A deposition on mRNAs.^59^ They further found that *Flacc* regulates *Drosophila* sex determination and dosage compensation through modulating *Sxl* alternative splicing.^59^ Guo *et al*. also identified the CG7358 gene (named as *Xio*) as a component of the *Drosophila* sex determination pathway, and further demonstrated its role in controlling *Sxl* alternative splicing via m^6^A methylation.^60^

### METTL16

The METTL3 homolog METTL16 (methyltransferase-like 16) controls cellular SAM level and installs m^6^A onto the U6 small nuclear RNA. METTL16 activity requires both the UACAGAGAA nonamer and a specific RNA structure.^6^

In addition to the above members, other subunits of the methyltransferase complex may exist to achieve precise post-transcriptional regulation through selectively recognizing candidate methylation sites.

## Demethylases/Erasers

Although METTL3 was discovered as an m^6^A writer several decades ago, the identity of demethylases remained a mystery until 2011, when Jia *et al*. unveiled that fat mass and obesity-associated protein (FTO) exhibits efficient m^6^A demethylase activity.^82^ Another m^6^A demethylase, α-ketoglutarate-dependent dioxygenase alkB homolog 5 (ALKBH5), was soon discovered in 2013,^17^ and was found to be highly expressed in the testes. The demonstration of their demethylase activity provided the first evidence of reversible post-transcriptional modification in mRNAs.

### FTO

FTO was originally reported as a demethylase for *N*^3^-methylthymidine in single-stranded DNA^83^ and for *N*^3^-methyluridine in single-stranded RNA^84^ in vitro. In 2011, Jia *et al*. found that FTO demethylates m^6^A in both DNA and RNA in vivo. Depletion of FTO induces significant increase in total m^6^A levels of polyadenylated RNA.^82^ As FTO oxidizes m^6^A to A, it generates *N*^6^-hydroxymethyladenosine (hm^6^A) as an intermediate product, and *N*^6^-formyladenosine (f^6^A) as a further oxidized product.^85^ The potential function of these oxidized labile intermediates needs further exploration. Intriguingly, a recent study demonstrated that FTO also displays demethylase activity towards m^6^A_m_ that is exclusively located at the first encoded nucleotide after the 7-methylguanosine cap structure of a large number of mRNAs and reduces the stability of m^6^A_m_-containing mRNAs in vivo,^86^ suggesting that FTO could work on multiple substrates. Hence, further studies are needed to reveal functional relevance of different FTO substrates.

FTO was first reported to be associated with increased body mass and obesity in human.^87–90^ It is widely expressed in all adult and fetal tissues and most highly expressed in the brain.^83^ FTO has been shown to function as an oncogene in both leukemia^91,92^ and glioblastoma.^76^ FTO expression is aberrantly upregulated by oncogenic proteins in certain subtypes of AML, where it promotes leukemogenesis and inhibits all-*trans*-retinoic acid (ATRA)-induced AML cell differentiation through reducing the m^6^A levels of a set of critical transcripts such as *ASB2* and *RARA*.^91^ A recent extended study further revealed that FTO efficiently demethylates internal m^6^A, and the inhibition of FTO by R-2HG leads to increased m^6^A levels in mRNAs, but only small changes in cap m^6^A_m_.^92^ Notably, specific m^6^A sites in FTO target mRNA transcripts such as *ASB2*, *RARA* and *MYC* have been demonstrated with quantitative methods (i.e., luciferase reporter/mutagenesis assays and gene region-speific m^6^A qPCR) to be regulated by FTO.^91,92^ The possible mechanisms underlying the phenomenon that both FTO and METTL14 play oncogenic roles in AML have been discussed.^77,93^

FTO promotes cell growth and self-renewal of human glioblastoma stem cells, and is required for substantial tumor progression. Its deficiency prolongs the lifespan of glioblastoma stem cell-grafted mice through regulating the expression of critical genes.^76^ Nevertheless, another study testing a number of different AML cell lines failed to observe an obvious effect of FTO on AML cell viability.^94^

Additionally, FTO regulates mouse pre-adipocyte differentiation by regulating alternative splicing of pre-mRNAs of genes involved in adipogenesis.^12^ Finally, a recent study revealed that depletion of FTO results in upregulation of terminal mRNA exons,^14^ suggesting that FTO also regulates alternative polyA site usage and 3’UTR processing.

### ALKBH5

As the second identified m^6^A demethylase, ALKBH5 displays m^6^A demethylation activity comparable to that of FTO. ALKBH5 may function in a sequence specific context, as it shows a preference for m^6^A within its consensus sequence rather than other methylated nucleotides in single-stranded RNA.^17^ ALKBH5 localizes to the nucleus and its depletion leads to global reduction of polyA RNAs in this cellular compartment,^17^ suggesting a role in the regulation of nuclear export of mRNA.

ALKBH5 is expressed in most tissues, being particularly abundant in the testes where it impacts mouse spermatogenesis and fertility.^17^ ALKBH5 regulates splicing and stability of mRNAs in the nuclei of spermatocytes and round spermatids by removing m^6^A from pre-mRNAs and allows the production of mRNAs containing longer 3’UTRs.^95^ Moreover, ALKBH5 plays an important role in the immune response to viral infections in macrophages. To inhibit interferon production, the nuclear protein DDX46 recruits ALKBH5 after viral infection to demethylate m^6^A-modified antiviral transcripts, thus sequestering them in the nucleus.^96^

ALKBH5 has also been shown to be important for cancer pathogenesis. A recent study revealed that overexpression of ALKBH5 is required for the proliferation and tumorigenesis of glioblastoma stem cells and predicts poor patient survival.^97^ Additionally, knockdown of ALKBH5 expression in MDA-MB-231 human breast cancer cells significantly reduced their capacity for tumor initiation attributing to the reduced of breast cancer stem cells.^98^

Given the varied distribution of the m^6^A demethylases across tissues and their essential roles in regulating m^6^A methylation, additional cell- or tissue-specific demethylases may exist to act on different RNA substrates.

## m^6^A readers

Chemical modifications can directly affect properties of RNA transcripts, including charge, base-pairing, secondary structure, and protein-RNA interactions, which in turn shape gene expression by modulating RNA processing, localization, translation, and eventually, decay.^1,3,4,99^ Prominently, m^6^A also indirectly affects RNA processing by recruiting specific reader proteins. To date, several m^6^A reader proteins have been identified in mammalian cellular extracts using combined approaches of affinity chromatography and mass spectrometry.^52^ Investigations about these reader proteins have begun to elucidate the ultimate role of m^6^A in RNA processing.

### Nuclear m^6^A readers

Several proteins selectively bind m^6^A-containing precursor RNAs in the nucleus. YTHDC1, the nuclear member of highly conserved YTH family proteins,^100^ locates in the nucleus and forms YT bodies at transcriptionally active sites adjacent to RNA processing speckles.^101^ Structural and binding studies revealed that YTHDC1 preferentially recognizes the GG(m^6^A)C sequences through its YTH domain.^102^

YTHDC1 promotes exon inclusion by selectively recruiting or inhibiting different splicing factors such as Serine-arginine repeat (SR) proteins.^7^ YTHDC1 also recognizes m^6^A on *XIST* and promotes *XIST*-mediated X chromosome silencing.^57^ In addition to its role in splicing regulation, very recent work in HeLa cells identified that YTHDC1 interacts with SRSF3 and nuclear RNA export factor 1 (NXF1) to facilitate the nuclear export of m^6^A-modified mRNAs,^18^ which expands the roles of YTHDC1-mediated m^6^A reading in mRNA metabolism regulation. In addition, YTHDC1 has been reported to be a potential tumor suppressor in endometrial cancer through its interaction with other splicing factors.^101,103^

Recent studies raised some controversies regarding whether the HNRNP family member HNRNPA2B1 is an m^6^A reader. Alarcon et al. reported that HNRNPA2B1 could directly bind m^6^A and regulate alternative splicing events and primary microRNA processing in concert with METTL3,^104^ whereas a structure-based study by Wu et al. revealed an “m^6^A switch” mechanism, instead of specific m^6^A binding, mediated by HNRNPA2B1.^105^ In addition, another two HNRNP proteins, HNRNPC^11^ and HNRNPG,^5^ function to regulate the processing of m^6^A-containing RNA transcripts. They do not bind to m^6^A directly, but instead, m^6^A serves as a structural switch to alter the RNA structure, which renders transcripts more accessible for binding by HNRNPC and HNRNPG.

### Cytoplasmic m^6^A readers

After being processed from precursor transcripts, mature mRNAs containing m^6^A are further regulated in the cytoplasm by other members of the YTH family: YTHDF1, YTHDF2, YTHDF3, and YTHDC2.^18,20,21,26,27,29,32,106,107^

YTHDF1 was initially demonstrated to bind methylated mRNA transcripts at sites near the stop codon, and its overall distribution pattern is similar to that of m^6^A sites on mRNAs. Mechanistic study has demonstrated that YTHDF1 interacts with the translation initiation machinery and enhances the translation efficiency of its target RNAs.^32^

YTHDF2 co-localizes with both deadenylation and decapping enzyme complexes under normal conditions and directs its targets to processing bodies.^21^ By directly recruiting the CCR4-NOT deadenylase complex, YTHDF2 accelerates the degradation of m^6^A-modified transcripts.^20^ Data were presented to show that YTHDF1 and YTHDF3 promote deadenylation through the CCR4-NOT complex to a lesser extent than YTHDF2.^20^ In zebrafish, *Ythdf2* deletion in embryos decelerates the decay of m^6^A-modified maternal mRNAs and impedes zygotic genome activation.^23^ Our recent study revealed that YTHDF2-mediated decay of transcripts of the arterial endothelial genes *notch1a* and *rhoca* contributes to the emergence of haematopoietic stem/progenitor cells (HSPCs) during endothelial-to-haematopoietic transition (EHT).^25^ In mice, YTHDF2 determines oocyte competence and early zygotic development by regulating maternal transcript dosage.^108^ In cells undergoing heat shock, YTHDF2 expression is increased, and its protein is translocated to the nucleus where YTHDF2 protects m^6^A residues in the 5’UTR of stress-induced transcripts from demethylation by FTO. Transcripts with enhanced 5’UTR methylation are selectively translated via a cap-independent mechanism.^29^

YTHDF3, together with YTHDF1, regulates mRNA translation by interacting with a common set of ribosomal proteins.^26,27^ In addition, YTHDF3 could also mediate mRNA decay by directly interacting with YTHDF2.^26^ Overall, YTHDF3 serves as a hub for fine-tuning the RNA accessibility of YTHDF1 and YTHDF2. YTHDF3 may also interact with other proteins to play additional cell-type specific roles on target transcripts.

YTHDC2, the largest member of the YTH family, also preferentially binds m^6^A within the consensus motif and can enhance the translation efficiency while decreasing the bundance of its target mRNAs.^106,107,109,110^ YTHDC2 has also been reported to play a role in spermatogenesis, in which it can interact with the meiosis-specific protein MEIOC to affect the stability of target transcripts during meiosis prophase I.^111,112^ Both male and female *Ythdc2* knockout mice display infertility, demonstrating defects in meiotic prophase I, suggesting its essential roles in spermatogenesis and oogenesis.^106^ Importantly, YTHDC2 is much larger than other YTH proteins (~160 kDa vs ~60 kDa), and contains multiple helicase domains and two Ankyrin repeats. These unique features may endow YTHDC2 with a variety of functions, including regulatory effects on RNA binding and RNA structure, and recruitment of or binding with other protein complex members.^106,113^ Intriguingly, a lack of m^6^A binding activity was reported by a CLIP study of YTHDC2 in HEK cells.^57,114^ The controversial findings that YTHDC2 acts as a reader of m^6^A in mouse testes but lacks m^6^A binding activity in HEK cells suggest a cell- or tissue-specific role of YTHDC2; it is also possible that YTHDC2 may indirectly regulate m^6^A-containing transcripts through interaction with other factors.

Methylated RNA-binding 1 (Mrb1), another YTH domain containing protein, has been reported to bind m^6^A in yeast.^31^ Additionally, several studies based on RNA pull-down approaches detected other m^6^A interactors, including ELAV like RNA binding protein 1 (ELAVL1, also known as HuR),^55,97,115^ FMR1,^116,117^ LRPPRC,^117^ and also IGF2BP family proteins.^24^ However, in most cases, whether these proteins directly bind to m^6^A or whether they are part of an m^6^A binding ribonucleoprotein complex needs further clarification.

## Functions in RNA metabolism

Recent accumulative studies revealed that m^6^A methylation regulates almost every aspect of mRNA metabolism, from expression and pre-mRNA processing in the nucleus to translation and mRNA decay in the cytoplasm.^1,3,4,10,118^ m^6^A has been reported to be associated with alternative polyadenylation (APA),^119,120^ which is coupled to the splicing of the last intron. m^6^A-involved regulation mediated by METTL3,^121^ ALKBH5^17^ and YTHDC1^18^ has been shown to modulate mRNA export from the nucleus to the cytoplasm. Several distinct mechanisms by which m^6^A promotes mRNA translation have been demonstrated including the YTHDF1-eIF3 pathway,^32^ the cap-independent translation^29,122^ and IGF2BPs-mediated translation.^24^ In the aspect of mRNA stability control, earlier studies generally considered m^6^A as a destabilizer facilitating mRNA degradation mainly through YTHDF2,^20,21,52,55^ while a recent study revealed a distinct function of m^6^A mediated by IGF2BP proteins in promoting the stability and storage of the mRNA targets.^24^ In addition, m^6^A could also alter RNA folding and structure,^5,11,28,73,123–127^ and sort transcripts into a fast track for mRNA metabolism.^7,18,21,26,27,32^ In the rest of this review, we focus on our current understanding of the roles of m^6^A in RNA splicing regulation and discuss the discrepancy and challenges in the field.

### m^6^A modulates RNA splicing

Processing of pre-mRNAs to mature mRNAs consists of three main steps: 5’capping, 3’polyadenylation and splicing. Splicing of the pre-mRNA, involving precise excision of introns and joining of exons in the nucleus,^128^ is an important process in gene expression and can increase the gene product diversity. Cis-regulatory RNA elements and trans-regulatory splicing factors participate in the process of alternative splicing regulation via specific interactions between trans-factors and cis-element sequences, which makes splicing regulation a dynamic and complicated process.^129^ Cis-regulatory RNA elements can be either intronic or exonic sequences and can promote (splicing enhancers) or inhibit (splicing silencers) splicing activity.^130^ Trans-regulatory splicing factors regulate splicing by forming spliceosomes with different categories of U snRNAs on pre-mRNAs and orchestrating with cis-regulatory elements.^131^ To date, exonic and intronic splicing enhancers (abbreviated as ESE and ISE, respectively) bound by the SR proteins of splicing activators as well as exonic and intronic splicing silencers (ESS and ISS, respectively) bound by the splicing repressor hnRNP proteins have been characterized and documented.^130^ In addition to RNA-protein interactions, RNA-RNA base pairing, chromatin modifications, small RNAs, and RNA polymerase II complex have also been reported to regulate alternative splicing patterns.^128,130^ Recently, several lines of evidence indicated that m^6^A also serves as an important pre-mRNA splicing regulator.

The predicted role of m^6^A as a splicing regulator is initially based on the early observations that m^6^A sites are concentrated in the introns of pre-mRNAs and were more abundant in pre-mRNAs than in mature mRNA.^132–134^ One previous study indicated that pre‑mRNAs are methylated at a level of ~4 m^6^A residues per mRNA, whereas for mature mRNAs, the number is ~2 m^6^A residues per mRNA,^132^ suggesting that methylation occurs in the nucleus and the removal of introns results in the loss of total m^6^A content per mRNA. Additionally, m^6^A is more likely to be found in introns and exons that undergo alternative splicing.^52^ Accumulating evidence from specific individual mRNAs supports the idea that m^6^A truly regulates splicing events, and its presence in either intronic regions or exonic regions plays important roles in alternative splicing.^6–13,52,118,124,135^

The localization of writers, readers and erasers of m^6^A to nuclear speckles, the locations for mRNA splicing and storage of splicing factors,^7,12,13,17,82^ also supports the link of m^6^A with splicing. Firstly, PAR-CLIP analysis showed that mRNAs undergoing alternative splicing have more METTL3 binding sites and *N*^6^-adenosine methylation sites.^13,52^ CLIP-seq of METTL3-METTL14 by another group revealed that 29-34% of their binding sites are in intronic regions.^54^ *Mettl3* depletion in mouse embryonic stem cells generally favors exon skipping and intron retention.^69^ A recent study also demonstrated that METTL3 functions in spermatogenesis by regulating the alternative splicing of spermatogonial differentiation and meiosis initiation- related mRNAs. *Mettl3* deletion led to aberrant splicing of important spermatogenesis-regulating genes, such as *Sohlh1* and *Dazl*, by altering the m^6^A modification of their transcripts.^46^ mRNAs that exhibit multiple isoforms due to alternative splicing are significantly more likely to contain m^6^A and be bound by METTL3 than mRNAs with only one spliced isoform.^13,52^ The m^6^A signaling-deficient Ime4/Yt521-B null mutant flies exhibit aberrant alternative splicing of *Sxl*.^8,9,15^ These results indicate that the recruitment of METTL3 to pre-mRNA is a co-transcriptional event with potential effects on either promoting or influencing splicing. WTAP is also considered as a splicing factor that binds to WT1 in *Drosophilia*,^136^ and its targets are mainly enriched in alternatively spliced exons rather than constitutively spliced exons.^13^ The finding that deficiency of WTAP results in altered mRNA isoforms also supports the idea that WTAP and m^6^A methylation affects mRNA splicing.^13^ Moreover, a recent study reported an interesting phenomenon that the m^6^A methyltransferase METTL16 regulates the expression of the SAM synthetase MAT2A by the enhanced splicing of a retained intron in the presence of its methylation substrate, a vertebrate conserved hairpin (hp1) in the MAT2A 3’UTR.^6^

Secondly, m^6^A erasers also affect splicing. Our previous report demonstrated that enhanced levels of m^6^A in response to FTO depletion in mouse pre-adipocytes promote the RNA binding ability of serine- and arginine-rich splicing factor 2 (SRSF2), leading to increased inclusion of target exons.^12^ A recent study in the human 293T cell line revealed that FTO binds pre-mRNAs in the nucleus and triggers inclusion of alternatively spliced exons. Upon FTO depletion, m^6^A was strongly enriched at intronic as well as exonic regions surrounding skipped exons, leading to exon skipping events.^14^ ALKBH5 deficiency also significantly influenced the nuclear speckle localization of several splicing factors and altered more than 3000 mRNA isoforms, suggesting its effects on splicing.^17^ A recent study further showed that ALKBH5-mediated m^6^A erasure in the nuclei of spermatocytes and round spermatids regulates splicing and stability of long 3’UTR mRNAs.^95^

Thirdly, m^6^A readers could also regulate splicing. Direct binding of m^6^A-decorated alternatively spliced exons by the m^6^A reader protein YTHDC1 facilitates their inclusion into mRNA. YTHDC1 promotes SRSF3 (driving exon inclusion) but antagonizes SRSF10 (driving exon exclusion) binding to mRNAs and affects mRNA splicing, leading to exon inclusion events.^7^ Moreover, YTHDC1-mediated m^6^A signaling plays a role in regulating the splicing of replication transcription activator (RTA) pre-mRNA encoded by the Kaposi’s sarcoma-associated herpesvirus (KSHV).^137^ HNRNP family proteins could affect splicing through forming ribonucleoprotein granules. Recent studies demonstrated that HNRNPA2B1 and HNRNPC are active splicing regulators correlated with m^6^A modification.^11,104^ Specially, HNRNPA2B1 has been reported to directly bind to m^6^A, leading to the regulation of alternative splicing events similar to those regulated by METTL3,^104^ as well as facilitating the processing of primary microRNAs (pri-miRNAs) to mature miRNAs.^104,135^ Additionally, m^6^A also regulates pre-mRNA processing by altering the local RNA structure to facilitate the binding of some HNRNP proteins, such as HNRNPC and HNRNPG.^5,11^

**Fig. 2:**
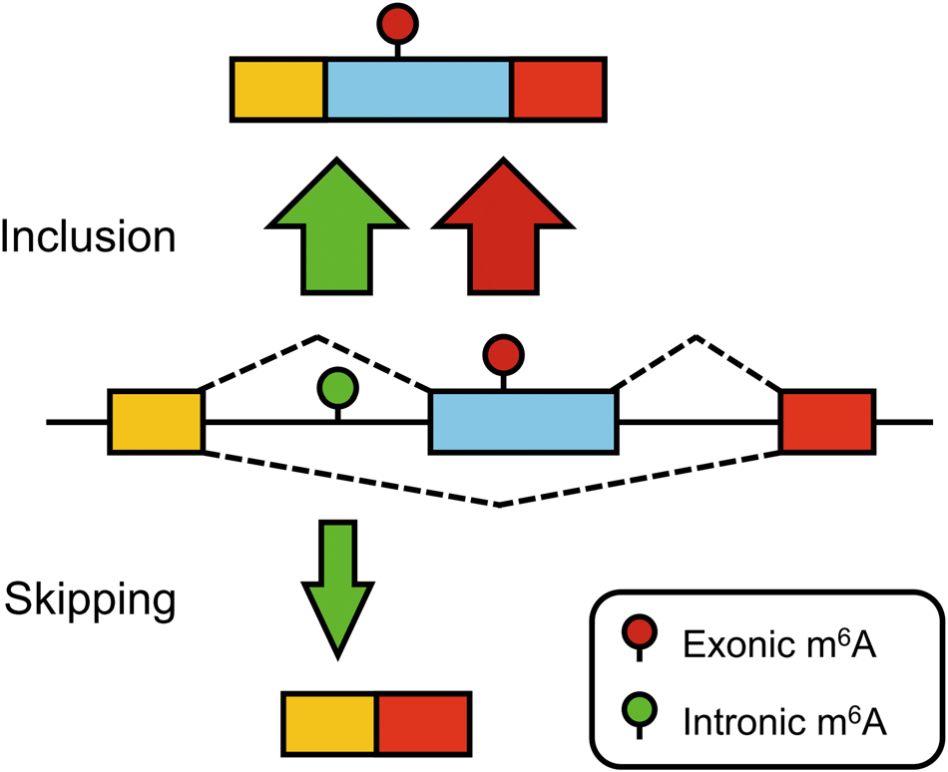
Schematic summary of the roles of m^6^A in regulating mRNA splicing. Exonic and most intronic m^6^A sites promotes exon inclusion while a small proportion of intronic m^6^A sites can also lead to exon skipping through a refined buffering system composed of its writers, readers, erasers as well as other splicing-related factors

Thus, m^6^A modification appears to change the mRNA isoform diversity by regulating alternative splicing. Based on the accumulating evidence that both exonic and intronic m^6^A sites regulate alternative splicing, we propose a model depicting that m^6^A modification serves as an elastic system in splicing regulation (Fig. 2), in which the rapid and dynamic changes in both levels and modification sites of m^6^A can elaborately regulate mRNA isoform dosage through the combined actions of m^6^A writers, readers and erasers: A, m^6^A sites located in alternatively spliced (AS) exons mainly lead to exon inclusion through m^6^A-dependent molecular mechanisms regulating alternative splicing. B, m^6^A sites buried into intronic regions could promote either exon inclusion or skipping.

Though many current reports support the association of m^6^A with RNA splicing regulation, one recent study from Ke et al.^138^ reported that the vast majority of exons harboring m^6^A in wild-type mouse stem cells are correctly spliced in cells lacking METTL3, suggesting that m^6^A is not obligatory for most of the splicing events. In their study, 99 exons from 2000 alternatively spliced cassette exons containing m^6^A were observed to have significantly different inclusion levels upon *Mettl3* depletion using Quantas software with FDR < 5% and ΔPSI ≥ 0.1. Ke et al. also examined the prevalence and distribution of m^6^A within intronic sequences by carrying out m^6^A-CLIP on pre-mRNAs and found that adenosine residues in introns have a smaller chance of being methylated compared with adenosine residues in exons.^138^ However, Liu et al. recently identified 39,060 m^6^A-switches among HNRNPC-binding sites and the majority (87%) of m^6^A-switches occur within introns.^11^ Pendleton et al. also found that most of the METTL16-dependent m^6^A peaks were in introns or spanned intron-exon boundaries.^6^

Several possibilities may explain different conclusions. The first possibility is that the analyses are done in different scales (at a global scale or with individual mRNAs). For the MBNL protein,^139^ a well-established splicing factor, only 6% of exons with MBNL binding clusters showed significant changes in splicing upon MBNL deficiency. This low proportion is attributed to the high-affinity binding of many RNA-binding proteins (RBPs) to thousands of different positions in the transcriptome, whereas probably only a subset of the interactions are truly associated with their specific functions.^140^ Considering that more and more m^6^A readers are being validated, m^6^A in different regions along mRNAs may play diverse regulatory roles at the post-transcriptional level, which can also explain why the splicing effect of m^6^A can be found on some specifically modified mRNAs but not all modified exons. Furthermore, not only exonic m^6^A on cassete exons but also m^6^A on flanking exons or introns may regulate alternative splicing, which is supported by the recent findings that many of the METTL3 binding sites are located in introns,^13,54^ illustrating that intronic sequences may be a major target of nuclear methylation. m^6^A may be introduced into introns at a relatively high level, which is then followed by rapid intron excision and degradation.

The current technical limitation in the quantifications of m^6^A modification might also result in different conclusions on whether m^6^A affects alternative splicing. Unlike m^5^C that can be directly quantified by C-to-T transition after bisufite treatment, m^6^A modification can only be quantified so far as the enrichment score by IP/input^51,52^ or detected as mutation or truncation products following a UV cross-linking procedure.^119,141,142^ The real methylation level cannot be estimated at the transcriptome level, thus it is hard to evaluate whether the effect on splicing is caused by relative m^6^A levels on specific sites. Besides, details of the methodology may also impact the sensitivity and efficiency of detection, such as sequencing depth, analysis pipeline and parameters used. Short reads and low converage result in less read-covered junctions.^143^ The boundaries of cutoff on inclusion change and FDR^139,144–146^ are quite different among labs and there is no gold standard for answering how many alternative splicing events can be considered as direct effects. All of these factors may have contributed to the controversy about the effect of m^6^A on splicing at the transcriptome level. Moreover, given the highly dynamic regulation of m^6^A by its methyltransferase and demethylases, potential removal of m^6^A in spliced exons cannot be ruled out. Thus, future systematic quantitative analysis of m^6^A in pre-mRNAs at single site resolution will be promising to provide a clearer view of the mechanism underlying m^6^A-mediated regulation of pre-mRNA splicing.

## Concluding remarks

Dynamic transcriptomic m^6^A modification is orchestrated by its writers and erasers, while functions of m^6^A in RNA metabolisms are carried out by its readers. Although some inconsistency in the current literature needs further detailed investigation, multiple lines of evidence support its significance in regulating both RNA metabolism and distinct biological processes. Recently, efforts have been made to directly detect m^6^A sites in RNA by engineering DNA polymerase mutant capable of increasing misincorporation opposite m^6^A^147^ or by using the nanopore technology for direct detection of RNA modifications.^148^ Developing the future direct sequencing technology for RNA m^6^A will be a key step to understand its dynamics and functions in vivo.

## References

[1] Roundtree IA, Evans ME, Pan T, He C (2017). Dynamic RNA modifications in gene expression regulation. Cell.

[2] Liu N, Pan T (2016). *N*^6^-methyladenosine-encoded epitranscriptomics. Nat. Struct. Mol. Biol..

[3] Zhao BS, Roundtree IA, He C (2017). Post-transcriptional gene regulation by mRNA modifications. Nat. Rev. Mol. Cell. Biol..

[4] Nachtergaele S, He C (2017). The emerging biology of RNA post-transcriptional modifications. RNA Biol..

[5] Liu N (2017). *N*^6^-methyladenosine alters RNA structure to regulate binding of a low-complexity protein. Nucleic Acids Res..

[6] Pendleton KE (2017). The U6 snRNA m^6^A Methyltransferase METTL16 regulates SAM synthetase intron retention. Cell.

[7] Xiao W (2016). Nuclear m^6^A reader YTHDC1 regulates mRNA splicing. Mol. Cell.

[8] Lence T (2016). m^6^A modulates neuronal functions and sex determination in *Drosophila*. Nature.

[9] Haussmann IU (2016). m^6^A potentiates *Sxl* alternative pre-mRNA splicing for robust *Drosophila* sex determination. Nature.

[10] Yang Y (2015). Dynamic m^6^A modification and its emerging regulatory role in mRNA splicing. Sci. Bull..

[11] Liu N (2015). *N*^6^-methyladenosine-dependent RNA structural switches regulate RNA-protein interactions. Nature.

[12] Zhao X (2014). FTO-dependent demethylation of *N*^6^-methyladenosine regulates mRNA splicing and is required for adipogenesis. Cell Res..

[13] Ping XL (2014). Mammalian WTAP is a regulatory subunit of the RNA *N*^6^-methyladenosine methyltransferase. Cell Res..

[14] Bartosovic M (2017). *N*^6^-methyladenosine demethylase FTO targets pre-mRNAs and regulates alternative splicing and 3’-end processing. Nucleic Acids Res..

[15] Kan L (2017). The m^6^A pathway facilitates sex determination in *Drosophila*. Nat. Commun..

[16] Yang X (2017). 5-methylcytosine promotes mRNA export - NSUN2 as the methyltransferase and ALYREF as an m^5^C reader. Cell Res..

[17] Zheng G (2013). ALKBH5 is a mammalian RNA demethylase that impacts RNA metabolism and mouse fertility. Mol. Cell.

[18] Roundtree IA (2017). YTHDC1 mediates nuclear export of *N*^6^-methyladenosine methylated mRNAs. eLife.

[19] Mishima Y, Tomari Y (2016). Codon usage and 3’ UTR length determine maternal mRNA stability in zebrafish. Mol. Cell.

[20] Du H (2016). YTHDF2 destabilizes m^6^A-containing RNA through direct recruitment of the CCR4-NOT deadenylase complex. Nat. Commun..

[21] Wang X (2014). *N*^6^-methyladenosine-dependent regulation of messenger RNA stability. Nature.

[22] Schwartz S (2014). Perturbation of m^6^A writers reveals two distinct classes of mRNA methylation at internal and 5’ sites. Cell Rep..

[23] Zhao BS (2017). m^6^A-dependent maternal mRNA clearance facilitates zebrafish maternal-to-zygotic transition. Nature.

[24] Huang H (2018). Recognition of RNA *N*^6^-methyladenosine by IGF2BP proteins enhances mRNA stability and translation. Nat. Cell. Biol..

[25] Zhang C (2017). m^6^A modulates haematopoietic stem and progenitor cell specification. Nature.

[26] Shi H (2017). YTHDF3 facilitates translation and decay of *N*^6^-methyladenosine-modified RNA. Cell Res..

[27] Li A (2017). Cytoplasmic m^6^A reader YTHDF3 promotes mRNA translation. Cell Res..

[28] Choi J (2016). *N*^6^-methyladenosine in mRNA disrupts tRNA selection and translation-elongation dynamics. Nat. Struct. Mol. Biol..

[29] Zhou J (2015). Dynamic m^6^A mRNA methylation directs translational control of heat shock response. Nature.

[30] Dominissini D (2016). The dynamic *N*^1^-methyladenosine methylome in eukaryotic messenger RNA. Nature.

[31] Bodi Z, Bottley A, Archer N, May ST, Fray RG (2015). Yeast m^6^A methylated mRNAs are enriched on translating ribosomes during meiosis, and under rapamycin treatment. PLoS ONE.

[32] Wang X (2015). *N*^6^-methyladenosine modulates messenger RNA translation efficiency. Cell.

[33] Clancy MJ, Shambaugh ME, Timpte CS, Bokar JA (2002). Induction of sporulation in *Saccharomyces cerevisiae* leads to the formation of *N*^6^-methyladenosine in mRNA: a potential mechanism for the activity of the *IME4* gene. Nucleic Acids Res..

[34] Shah JC, Clancy MJ (1992). IME4, a gene that mediates MAT and nutritional control of meiosis in S*accharomyces cerevisiae*. Mol. Cell. Biol..

[35] Bodi Z, Button JD, Grierson D, Fray RG (2010). Yeast targets for mRNA methylation. Nucleic Acids Res..

[36] Zhong SL (2008). MTA is an *Arabidopsis* messenger RNA adenosine methylase and interacts with a homolog of a sex-specific splicing factor. Plant Cell.

[37] Haugland RA, Cline MG (1980). Post-transcriptional modifications of oat coleoptile ribonucleic acids. 5’-terminal capping and methylation of internal nucleosides in poly(A)-rich RNA. Eur. J. Biochem..

[38] Kennedy TD, Lane BG (1979). Wheat embryo ribonucleates. XIII. Methyl-substituted nucleoside constituents and 5’-terminal dinucleotide sequences in bulk poly(AR)-rich RNA from imbibing wheat embryos. Can. J. Biochem..

[39] Hongay CF, Orr-Weaver TL (2011). *Drosophila* Inducer of MEiosis 4 (IME4) is required for Notch signaling during oogenesis. Proc. Natl Acad. Sci. USA.

[40] Yoon KJ (2017). Temporal control of mammalian cortical neurogenesis by m^6^A methylation. Cell.

[41] Bansal H (2014). WTAP is a novel oncogenic protein in acute myeloid leukemia. Leukemia.

[42] Schibler U, Kelley DE, Perry RP (1977). Comparison of methylated sequences in messenger RNA and heterogeneous nuclear RNA from mouse L cells. J. Mol. Biol..

[43] Wei CM, Moss B (1977). Nucleotide sequences at the *N*^6^-methyladenosine sites of HeLa cell messenger ribonucleic acid. Biochemistry.

[44] Desrosiers R, Friderici K, Rottman F (1974). Identification of methylated nucleosides in messenger RNA from Novikoff hepatoma cells. Proc. Natl Acad. Sci. USA.

[45] Horowitz S, Horowitz A, Nilsen TW, Munns TW, Rottman FM (1984). Mapping of *N*^6^-methyladenosine residues in bovine prolactin mRNA. Proc. Natl Acad. Sci. USA.

[46] Xu K (2017). Mettl3-mediated m^6^A regulates spermatogonial differentiation and meiosis initiation. Cell Res..

[47] Chen T (2015). m^6^A RNA methylation is regulated by microRNAs and promotes reprogramming to pluripotency. Cell Stem Cell.

[48] Lavi S, Shatkin AJ (1975). Methylated simian virus 40-specific RNA from nuclei and cytoplasm of infected BSC-1 cells. Proc. Natl Acad. Sci. USA.

[49] Furuichi Y (1975). Methylated, blocked 5 termini in HeLa cell mRNA. Proc. Natl Acad. Sci. USA.

[50] Adams JM, Cory S (1975). Modified nucleosides and bizarre 5’-termini in mouse myeloma mRNA. Nature.

[51] Meyer KD (2012). Comprehensive analysis of mRNA methylation reveals enrichment in 3’ UTRs and near stop codons. Cell.

[52] Dominissini D (2012). Topology of the human and mouse m^6^A RNA methylomes revealed by m^6^A-seq. Nature.

[53] Bokar JA, Shambaugh ME, Polayes D, Matera AG, Rottman FM (1997). Purification and cDNA cloning of the AdoMet-binding subunit of the human mRNA (*N*^6^-adenosine)-methyltransferase. RNA.

[54] Liu J (2014). A METTL3-METTL14 complex mediates mammalian nuclear RNA *N*^6^-adenosine methylation. Nat. Chem. Biol..

[55] Wang Y (2014). *N*^6^-methyladenosine modification destabilizes developmental regulators in embryonic stem cells. Nat. Cell. Biol..

[56] Agarwala SD, Blitzblau HG, Hochwagen A, Fink GR (2012). RNA methylation by the MIS complex regulates a cell fate decision in yeast. PLoS Genet..

[57] Patil DP (2016). m^6^A RNA methylation promotes *XIST*-mediated transcriptional repression. Nature.

[58] Wen J (2018). Zc3h13 regulates nuclear RNA m^6^A methylation and mouse embryonic stem cell self-renewal. Mol. Cell.

[59] Knuckles P (2018). Zc3h13/Flacc is required for adenosine methylation by bridging the mRNA-binding factor Rbm15/Spenito to the m^6^A machinery component Wtap/Fl(2)d. Genes Dev..

[60] Guo, J., Tang, H. W., Li, J., Perrimon, N. & Yan D. Xio is a component of the *Drosophila* sex determination pathway and RNA *N*^6^-methyladenosine methyltransferase complex. *Proc. Natl Acad. Sci. USA* **115**, 3674–3679 (2018). 10.1073/pnas.1720945115 PMC5889661 29555755

[61] Śledź P, Jinek M (2016). Structural insights into the molecular mechanism of the m^6^A writer complex. eLife.

[62] Wang P, Doxtader KA, Nam Y (2016). Structural basis for cooperative function of Mettl3 and Mettl14 methyltransferases. Mol. Cell.

[63] Wang X (2016). Structural basis of *N*^6^-adenosine methylation by the METTL3–METTL14 complex. Nature.

[64] Rottman FM, Bokar JA, Narayan P, Shambaugh ME, Ludwiczak R (1994). *N*^6^-adenosine methylation in mRNA: substrate specificity and enzyme complexity. Biochimie.

[65] Harper JE, Miceli SM, Roberts RJ, Manley JL (1990). Sequence specificity of the human mRNA *N*^6^-adenosine methylase *in vitro*. Nucleic Acids Res..

[66] Lin S, Choe J, Du P, Triboulet R, Gregory RI (2016). The m^6^A methyltransferase METTL3 promotes translation in human cancer cells. Mol. Cell.

[67] Bodi Z (2012). Adenosine methylation in *Arabidopsis* mRNA is associated with the 3’ end and reduced levels cause developmental defects. Front. Plant Sci..

[68] Aguilo F (2015). Coordination of m^6^A mRNA methylation and gene transcription by ZFP217 regulates pluripotency and reprogramming. Cell Stem Cell.

[69] Geula S (2015). m^6^A mRNA methylation facilitates resolution of naïve pluripotency toward differentiation. Science.

[70] Li HB (2017). m^6^A mRNA methylation controls T cell homeostasis by targeting the IL-7/STAT5/SOCS pathways. Nature.

[71] Lv J (2018). Endothelial-specific m^6^A modulates mouse hematopoietic stem and progenitor cell development via Notch signaling. Cell Res..

[72] Bujnicki JM, Feder M, Radlinska M, Blumenthal RM (2002). Structure prediction and phylogenetic analysis of a functionally diverse family of proteins homologous to the MT-A70 subunit of the human mRNA: m^6^A methyltransferase. J. Mol. Evol..

[73] Schwartz S (2013). High-resolution mapping reveals a conserved, widespread, dynamic mRNA methylation program in yeast meiosis. Cell.

[74] Huang, J. et al. Solution structure of the RNA recognition domain of METTL3-METTL14 *N*^6^-methyladenosine methyltransferase. *Protein Cell*10.1007/s13238-018-0518-7 (2018). 10.1007/s13238-018-0518-7 PMC6418081 29542011

[75] Lin Z (2017). Mettl3-/Mettl14-mediated mRNA *N*^6^-methyladenosine modulates murine spermatogenesis. Cell Res..

[76] Cui Q (2017). m^6^A RNA methylation regulates the self-renewal and tumorigenesis of glioblastoma stem cells. Cell Rep..

[77] Weng H (2018). METTL14 inhibits hematopoietic stem/progenitor differentiation and promotes leukemogenesis via mRNA m^6^A modification. Cell Stem Cell.

[78] Horiuchi K (2013). Identification of Wilms’ tumor 1-associating protein complex and its role in alternative splicing and the cell cycle. J. Biol. Chem..

[79] Granadino B, Campuzano S, Sanchez L (1990). The *Drosophila melanogaster* fl(2)d gene is needed for the female-specific splicing of Sex-lethal RNA. EMBO J..

[80] Ortega A (2003). Biochemical function of female-lethal (2)D/Wilms’ tumor suppressor-1-associated proteins in alternative pre-mRNA splicing. J. Biol. Chem..

[81] Yue Y (2018). VIRMA mediates preferential m^6^A mRNA methylation in 3’UTR and near stop codon and associates with alternative polyadenylation. Cell Discov..

[82] Jia G (2011). *N*^6^-methyladenosine in nuclear RNA is a major substrate of the obesity-associated FTO. Nat. Chem. Biol..

[83] Gerken T (2007). The obesity-associated FTO gene encodes a 2-oxoglutarate-dependent nucleic acid demethylase. Science.

[84] Jia G (2008). Oxidative demethylation of 3-methylthymine and 3-methyluracil in single-stranded DNA and RNA by mouse and human FTO. FEBS Lett..

[85] Fu Y (2013). FTO-mediated formation of *N*^6^-hydroxymethyladenosine and *N*^6^-formyladenosine in mammalian RNA. Nat. Commun..

[86] Mauer J (2017). Reversible methylation of m^6^A_m_ in the 5’ cap controls mRNA stability. Nature.

[87] Dina C (2007). Variation in *FTO* contributes to childhood obesity and severe adult obesity. Nat. Genet..

[88] Frayling TM (2007). A common variant in the *FTO* gene is associated with body mass index and predisposes to childhood and adult obesity. Science.

[89] Scuteri A (2007). Genome-wide association scan shows genetic variants in the *FTO* gene are associated with obesity-related traits. PLoS Genet..

[90] Zhao X, Yang Y, Sun BF, Zhao YL, Yang YG (2014). FTO and obesity: mechanisms of association. Curr. Diab. Rep..

[91] Li Z (2017). FTO plays an oncogenic role in acute myeloid leukemia as a *N*^6^-methyladenosine RNA demethylase. Cancer Cell..

[92] Su R (2018). R-2HG exhibits anti-tumor activity by targeting FTO/m^6^A/MYC/CEBPA Signaling. Cell.

[93] Deng X (2017). Role of *N*^6^-methyladenosine modification in cancer. Curr. Opin. Genet. Dev..

[94] Vu LP (2017). The *N*^6^-methyladenosine (m^6^A)-forming enzyme METTL3 controls myeloid differentiation of normal hematopoietic and leukemia cells. Nat. Med..

[95] Tang C (2018). ALKBH5-dependent m^6^A demethylation controls splicing and stability of long 3’-UTR mRNAs in male germ cells. Proc. Natl Acad. Sci. USA.

[96] Zheng Q, Hou J, Zhou Y, Li Z, Cao X (2017). The RNA helicase DDX46 inhibits innate immunity by entrapping m^6^A-demethylated antiviral transcripts in the nucleus. Nat. Immunol..

[97] Zhang S (2017). m^6^A demethylase ALKBH5 maintains tumorigenicity of glioblastoma stem-like cells by sustaining FOXM1 expression and cell proliferation program. Cancer Cell..

[98] Zhang C (2016). Hypoxia-inducible factors regulate pluripotency factor expression by ZNF217- and ALKBH5-mediated modulation of RNA methylation in breast cancer cells. Oncotarget.

[99] Liu N, Pan T (2016). *N*^6^-methyladenosine-encoded epitranscriptomics. Nat. Struct. Mol. Biol..

[100] Zhang Z (2010). The YTH domain is a novel RNA binding domain. J. Biol. Chem..

[101] Zhang B (2010). Alternative splicing-related factor YT521: an independent prognostic factor in endometrial cancer. Int. J. Gynecol. Cancer.

[102] Xu C (2014). Structural basis for selective binding of m^6^A RNA by the YTHDC1 YTH domain. Nat. Chem. Biol..

[103] Hirschfeld M (2014). Hypoxia-dependent mRNA expression pattern of splicing factor YT521 and its impact on oncological important target gene expression. Mol. Carcinog..

[104] Alarcón CR (2015). HNRNPA2B1 is a mediator of m^6^A -dependent nuclear RNA processing events. Cell.

[105] Wu B (2018). Molecular basis for the specific and multivariant recognitions of RNA substrates by human hnRNP A2/B1. Nat. Commun..

[106] Hsu PJ (2017). Ythdc2 is an *N*^6^-methyladenosine binding protein that regulates mammalian spermatogenesis. Cell Res..

[107] Bailey AS (2017). The conserved RNA helicase YTHDC2 regulates the transition from proliferation to differentiation in the germline. eLife.

[108] Ivanova I (2017). The RNA m^6^A reader YTHDF2 is essential for the post-transcriptional regulation of the maternal transcriptome and oocyte competence. Mol. Cell.

[109] Wojtas MN (2017). Regulation of m^6^A transcripts by the 3’ → 5’ RNA helicase YTHDC2 is essential for a successful meiotic program in the mammalian germline. Mol. Cell.

[110] Jain D (2018). ketu mutant mice uncover an essential meiotic function for the ancient RNA helicase YTHDC2. eLife.

[111] Abby E (2016). Implementation of meiosis prophase I programme requires a conserved retinoid-independent stabilizer of meiotic transcripts. Nat. Commun..

[112] Soh YQS (2017). Meioc maintains an extended meiotic prophase I in mice. PLoS Genet..

[113] Tanabe A (2016). RNA helicase YTHDC2 promotes cancer metastasis via the enhancement of the efficiency by which *HIF-1α* mRNA is translated. Cancer Lett..

[114] Patil DP, Pickering BF, Jaffrey SR (2018). Reading m^6^A in the transcriptome: m^6^A-binding proteins. Trends Cell. Biol..

[115] Hosono Y (2017). Oncogenic role of *THOR*, a conserved cancer/testis long non-coding RNA. Cell.

[116] Edupuganti RR (2017). *N*^6^-methyladenosine (m^6^A) recruits and repels proteins to regulate mRNA homeostasis. Nat. Struct. Mol. Biol..

[117] Arguello AE, DeLiberto AN, Kleiner RE (2017). RNA chemical proteomics reveals the *N*^6^-methyladenosine (m^6^A)-regulated protein-RNA interactome. J. Am. Chem. Soc..

[118] Adhikari S, Xiao W, Zhao YL, Yang YG (2016). m^6^A: Signaling for mRNA splicing. RNA Biol..

[119] Ke S (2015). A majority of m^6^A residues are in the last exons, allowing the potential for 3’ UTR regulation. Genes Dev..

[120] Molinie B (2016). m^6^A-LAIC-seq reveals the census and complexity of the m^6^A epitranscriptome. Nat. Methods.

[121] Fustin JM (2013). RNA-methylation-dependent RNA processing controls the speed of the circadian clock. Cell.

[122] Meyer KD (2015). 5’UTR m^6^A promotes cap-independent translation. Cell.

[123] Batista PJ (2014). m^6^A RNA modification controls cell fate transition in mammalian embryonic stem cells. Cell Stem Cell.

[124] Roost C (2015). Structure and thermodynamics of *N*^6^-methyladenosine in RNA: a spring-loaded base modification. J. Am. Chem. Soc..

[125] Spitale RC (2015). Structural imprints i*n vivo* decode RNA regulatory mechanisms. Nature.

[126] Wan Y (2014). Landscape and variation of RNA secondary structure across the human transcriptome. Nature.

[127] Zhou KI (2016). *N*^6^-methyladenosine modification in a long noncoding RNA hairpin predisposes its conformation to protein binding. J. Mol. Biol..

[128] Baralle FE, Giudice J (2017). Alternative splicing as a regulator of development and tissue identity. Nat. Rev. Mol. Cell. Biol..

[129] Braunschweig U, Gueroussov S, Plocik AM, Graveley BR, Blencowe BJ (2013). Dynamic integration of splicing within gene regulatory pathways. Cell.

[130] Lee Y, Rio DC (2015). Mechanisms and regulation of alternative pre-mRNA splicing. Annu. Rev. Biochem..

[131] Wahl MC, Will CL, Lührmann R (2009). The spliceosome: design principles of a dynamic RNP machine. Cell.

[132] Salditt-Georgieff M (1976). Methyl labeling of HeLa cell hnRNA: a comparison with mRNA. Cell.

[133] Carroll SM, Narayan P, Rottman FM (1990). *N*^6^-methyladenosine residues in an intron-specific region of prolactin pre-mRNA. Mol. Cell. Biol..

[134] Stoltzfus CM, Dane RW (1982). Accumulation of spliced avian retrovirus messenger-rna is inhibited in S-adenosylmethionine-depleted chicken-embryo fibroblasts. J. Virol..

[135] Alarcón CR, Lee H, Goodarzi H, Halberg N, Tavazoie SF (2015). *N*^6^-methyladenosine marks primary microRNAs for processing. Nature.

[136] Penn JK (2008). Functioning of the *Drosophila* Wilms’-tumor-1-associated protein homolog, Fl(2)d, in sex-lethal-dependent alternative splicing. Genetics.

[137] Ye F, Chen ER, Nilsen TW (2017). Kaposi’s sarcoma-associated herpesvirus utilizes and manipulates RNA *N*^6^-Adenosine methylation to promote lytic replication. J. Virol..

[138] Ke S (2017). m^6^A mRNA modifications are deposited in nascent pre-mRNA and are not required for splicing but do specify cytoplasmic turnover. Genes Dev..

[139] Wang ET (2012). Transcriptome-wide regulation of pre-mRNA splicing and mRNA localization by muscleblind proteins. Cell.

[140] König J (2010). iCLIP reveals the function of hnRNP particles in splicing at individual nucleotide resolution. Nat. Struct. Mol. Biol..

[141] Chen K (2015). High-resolution *N*^6^-methyladenosine m^6^A map using photo-crosslinking-assisted m^6^A sequencing. Angew. Chem. Int. Ed. Engl..

[142] Linder B (2015). Single-nucleotide-resolution mapping of m^6^A and m^6^Am throughout the transcriptome. Nat. Methods.

[143] Norton S, Vaquero-Garcia J, Lahens NF, Grant GR, Barash FC (2018). Outlier detection for improved differential splicing quantification from RNA-Seq experiments with replicates. Bioinformatics.

[144] Goodwin M (2015). MBNL sequestration by toxic RNAs and RNA misprocessing in the myotonic dystrophy brain. Cell Rep..

[145] Charizanis K (2012). Muscleblind-like 2-mediated alternative splicing in the developing brain and dysregulation in myotonic dystrophy. Neuron.

[146] Taliaferro JM (2016). Distal alternative last exons localize mRNAs to neural projections. Mol. Cell.

[147] Aschenbrenner J (2017). Engineering of a DNA polymerase for direct m^6^A sequencing. Angew. Chem. Int. Ed. Engl..

[148] Garalde DR (2018). Highly parallel direct RNA sequencing on an array of nanopores. Nat. Methods.

